# Epidemiological survey of ticks and tick-borne pathogens in pet dogs in south-eastern China

**DOI:** 10.1051/parasite/2017036

**Published:** 2017-10-03

**Authors:** Jianwei Zhang, Qingbiao Liu, Demou Wang, Wanmeng Li, Frédéric Beugnet, Jinlin Zhou

**Affiliations:** 1 Merial International Trade (Shanghai) Co. Ltd., Shanghai 200040 PR China; 2 Shanghai Veterinary Research Institute, Chinese Academy of Agricultural Sciences No. 518, Ziyue Road, Minhang District, Shanghai 200241 PR China; 3 Merial SAS, 6900 7 Lyon France

**Keywords:** Ticks, tick-borne pathogens, pet dogs, south-eastern China, epidemiological survey

## Abstract

To understand the epidemiology of tick infestation and tick-borne diseases in pet dogs in south-eastern China and to develop a reference for their prevention and treatment, we collected 1550 ticks parasitizing 562 dogs in 122 veterinary clinics from 20 cities of south-eastern China. Dogs were tested for common tick-borne pathogens; collected ticks were identified and processed for the detection of tick-borne pathogens. The use of an *in vitro* ELISA diagnostic kit for antibody detection (SNAP®4Dx® Plus) on dog sera found the infection rates with *Borrelia* *burgdorferi* sensu lato, *Ehrlichia canis,* and *Anaplasma* spp. to be 0.4%, 1.3% and 2.7%, respectively. By using a specific ELISA method, the infection rate with *Babesia gibsoni* was 3.9%. *Rhipicephalus sanguineus* sensu lato, *Haemaphysalis longicornis* and *Rhipicephalus haemaphysaloides* were the major tick species identified on pet dogs. PCR tests were conducted to detect five tick-borne pathogens in 617 ticks. The infection rate was 10.2% for *E. canis*, 3.4% for *Anaplasma platys*, 2.3% for *B. gibsoni*, 0.3% for *B. burgdorferi* s.l. and 0% for *Babesia canis*. Some ticks were co-infected with two (1.46%) or three pathogens (0.16%). These results indicate the infestation of pet dogs by ticks infected with tick-borne pathogens in south-eastern China, and the need for effective treatment and routine prevention of tick infestations in dogs.

## Abstract (fr)

Pour comprendre l'épidémiologie de l'infestation par les tiques et les maladies transmises par les tiques chez les chiens domestiques dans le Sud-Est de la Chine et afin de fournir une référence pour leur prévention et leur traitement, nous avons collecté 1550 tiques parasitant 562 chiens dans 122 cliniques vétérinaires de 20 villes du Sud-Est de la Chine. Les chiens ont été testés pour des agents pathogènes courants de tiques. Les tiques collectées ont été identifiées et traitées pour la détection de pathogènes transmissibles par tiques. L'utilisation d'un kit de diagnostic *in vitro* ELISA pour la détection d'anticorps (SNAP®4Dx® Plus) sur les sérums de chiens, a mesuré le taux d'infection de *Borrelia burgdorferi sensu lato*, *Ehrlichia canis* et *Anaplasma* spp. à 0,4 %, 1,3 % et 2,7 % respectivement. En utilisant une méthode ELISA spécifique, le taux d'infection de *Babesia gibsoni* était de 3,9 %. *Rhipicephalus sanguineus sensu lato*, *Haemaphysalis longicornis* et *Rhipicephalus haemaphysaloides* étaient les principales espèces de tiques identifiées sur les chiens de compagnie. Des tests par PCR ont été effectués pour détecter cinq agents pathogènes transmissibles par les tiques dans 617 tiques. Le taux d'infection était de 10,2 % pour *E. canis*, 3,4 % pour *Anaplasma platys*, 2,3 % pour *B. gibsoni*, 0,3 % pour *B. burgdorferi s.l.* et 0 % pour *Babesia canis*. Certaines tiques étaient co-infectées avec deux (1,46 %) ou trois agents pathogènes (0,16 %). Ces résultats indiquent l'infestation des chiens de compagnie par des tiques infectées par des agents pathogènes transmissibles par les tiques dans le Sud-Est de la Chine et la nécessité d'un traitement efficace et de la prévention systématique des infestations de tiques chez les chiens.

## Introduction

The number of pet dogs is increasing in China as living standards have improved. As in many other countries, the dog has become a bonded family member. Among canine diseases, the zoonotic diseases are of significant importance in public health [1,2]. Ticks are one of the most common ectoparasites in dogs and are involved in the transmission of a number of major diseases in both dogs and humans [3,4]. With climate and environmental changes, as well as the appearance of new and re-emerging tick-borne diseases, ticks have been the focus of extensive attention in recent years [5,6]. The increase in the pet dog population and their close relationship with humans in China has created the need for research into the epidemiological status of ticks and the pathogens they transmit to pet dogs. However, there is very little reliable information on ticks and tick-borne agents in dogs in China. Dominant ticks reported in dogs in China are *Rhipicephalus sanguineus*, *Haemaphysalis longicornis* and *Rhipicephalus haemaphysaloides* [7,8]; the common tick-borne agents found in dogs in China included *Ehrlichia canis*, *Babesia gibsoni,* and *Anaplasma* species [7,9,10,11]. A survey of the occurrence of *Borrelia* *burgdorferi* sensu lato, *Ehrlichia canis*, and *Anaplasma phagocytophilum* in dogs was undertaken and found the seroprevalence to be 0.17%, 2.17% and 0.5%, respectively [10]. A serological investigation of vector-borne diseases in dogs from rural areas of China has shown the seroprevalence of *A. phagocytophilum* to be 7.7% by the SNAP 4Dx test kit, and 50% by indirect fluorescent antibody (IFA) testing [11]. A 3.47% seroprevalence of *Babesia gibsoni* in pet dogs was observed in East China [7]. Recently, molecular detection has indicated mixed infections with tick-borne *Anaplasma* species in dogs in Henan, China [9] and *Ehrlichia canis*, and *Babesia* spp. in dogs in some cities of China [8]. Since epidemiological surveys on ticks and their transmitted diseases in dogs in China are scarce, there is a need for data that are more comprehensive in their coverage of the region. Therefore, we carried out a broader epidemiological survey covering south-eastern China that included 122 veterinary clinics to confirm and expand on the data reported to date.

## Materials and methods

### Ethics approval

The experimental animals in tick feeding were treated following the approved guidelines from the Animal Care and Use Committee of the Shanghai Veterinary Research Institute. Sampling procedures also complied with these guidelines.

### Collection and handling of serum samples

Twenty cities in 16 provinces in south-eastern China were selected between October and November 2013. Three to five pet clinics were taken as sampling sites for each city. Five to 10 blood samples were collected from dogs at each clinic (0.5–1 mL). Dogs were presented for reasons unrelated to the suspicion of canine vector-borne disease. Collected serum was stored at −30 °C prior to testing. Each sample was registered and numbered.

### Collection and handling of tick samples

Dogs were examined at presentation and a sample of ticks was collected from each dog if blood was sampled. No more than 10 ticks were collected from each dog and placed in a collection tube containing a wet cotton ball. Each sample was registered and numbered.

### Testing for the infection rate to tick-borne pathogens in dogs

#### Testing for *Ehrlichia*, *Anaplasma*, and *Borrelia* infection rates

Serum samples from pet dogs were tested for antibodies by the rapid in-clinic enzyme-linked immunosorbent assay (ELISA) kit (SNAP^®^ 4Dx^®^, IDEXX Laboratories, Westbrook, Maine, USA), according to the instructions in the product package. Briefly, a 150 μL serum sample was taken and placed in one reaction tube, 200 μL testing reagent was added and after mixing the sample was put into the device sample well.

#### Serological detection of *Babesia gibsoni*

An enzyme-linked immunosorbent assay (ELISA) used for *Babesia gibsoni* was specifically developed in accordance with the established method [12]. The antigen used was recombinant *B. gibsoni* BgTRAP, expressed in *Escherichia coli*. A positive serum sample from an experimentally infected dog and negative control dog serum were sourced from the Shanghai Veterinary Research Institute, Chinese Academy of Agricultural Sciences.

#### Identification of parasitic ticks on dogs

In accordance with the morphology of ticks, an observation was performed microscopically to determine their developmental stage (larval, nymph, adult) and species. Ticks were identified using recognized morphological keys [13,14]. Larval and nymphal stages that were present were developed to the adult stage for identification through animal laboratory feeding.

### Testing for tick-borne pathogens

#### Extraction of tick DNA

Following morphological identification, 3 to 5 ticks from each infested dog were processed for the extraction of pathogen DNA. A single tick was placed in liquid nitrogen and finely ground. A genomic DNA extraction kit was used (QIAamp DNA Mini kit, Qiagen, Hilden, Germany). A nucleic acid detector was used to assess the concentration and content of the genomic DNA.

#### Polymerase chain reaction (PCR)

PCR technology was used for the detection of pathogens in ticks, in combination with DNA sequencing for precise determination of pathogens. The target gene, primer, reaction conditions by PCR, and references for each pathogen are provided in Table 1.

**Table 1:** Overview of the target gene, primer and PCR methods used for pathogen identification in sampled ticks.

Pathogen	Target gene	Primer sequence (5'–3')	Method	Reference
*Ehrlichia canis/ Anaplasma platys*	16S rRNA gene	Outer primer F: AGAGTTTGATCCTGGCTCAG Outer primer R: TAGCACTCATCGTTTACAGC Nested Primer: *A. platys*-specific primers F: AAGTCGAACGGATTTTGTC, and Primer R: CTTTAACTTACCGAACC *E.canis*- specific primers F: CAATTATTTATAGCCTCTGGCTATAGGA, and Primer R: GAGTTTGCCGGGACTTCTTCT	Nested PCR	[33]
*Babesia gibsoni/ Babesia canis*	18S rRNA gene	PIRO-A: AGGGAGCCTGAGAGACGGCTACC PIRO-B: TTAAATACGAATGCCCCCAAC	PCR	[34]
*Borrelia burgdorferi* sensu lato	Flagellin gene	Outer primer F: TGGTATGGGAGTTTCTGG Outer primer R: TCTGTCATTGTAGCATCTTT Nested primer F: CAGACAACAGAGGGAAAT Nested primer R:TCAAGTCTATTTTGGAAAGCACC	Nested PCR	[35]

#### Statistical analysis

Differences in the positive rates of pathogens in different tick species were tested by Chi-square, which was performed using IBM SPSS Statistics 20.0 software. A probability *p* value < 0.05 was considered statistically significant.

## Results

### Sample collection

**Figure 1:**
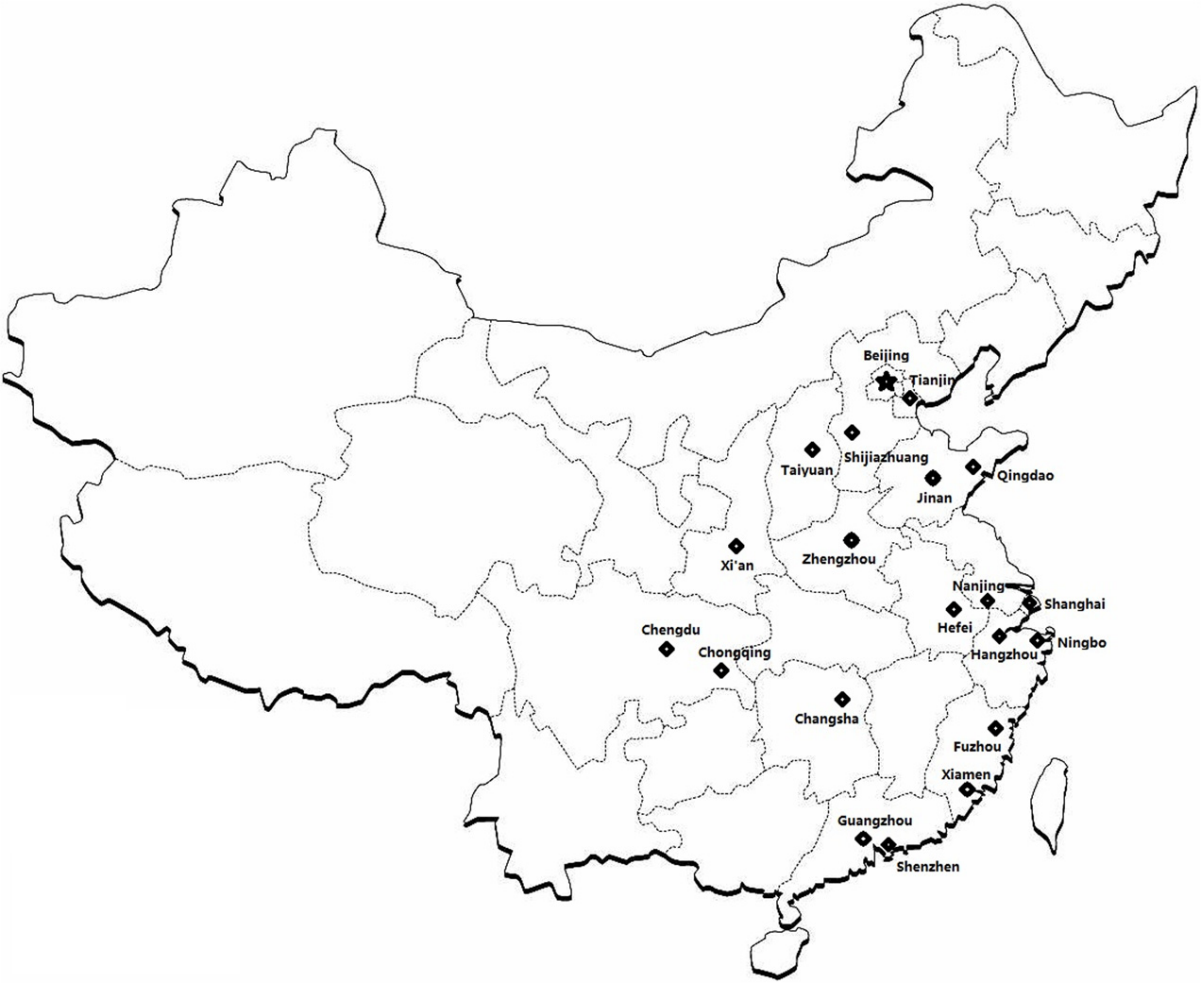
Location of 20 large cities in China selected for sampling.

Samples were collected in 20 large cities (Figure 1), from 16 provinces and municipalities directly under the Central Government in the Central and Eastern region of China. A total of 562 canine sera and 1550 ticks infesting dogs were collected and respectively tested or morphologically identified, while 617 tick DNA samples were prepared. The numbers of canine serum samples ranged from 6 to 57 in each city, 0 to 278 ticks were collected and 0 to 133 tick DNA samples were prepared.

### Dog serological tests

The results of the 526 serological tests are presented in Table 2. Overall, there were 2 cases of *Borrelia* infection (infection rate 0.38%), 7 cases of *Ehrlichia* infection (1.33%), 14 cases of *Anaplasma* infection (2.66%), 1 case of heartworm (*Dirofilaria immitis*) infection (0.19%) and 22 cases of *B. gibsoni* infection (3.91%). No co-infected samples were found. *B. gibsoni* infection was the most frequently detected among these tests.

**Table 2:** Serological positivity for *Anaplasma* spp., *Borrelia* spp., *Ehrlichia* spp. and *Babesia gibsoni* infection in pet dogs by ELISA.

Sample		Borrelia spp.	Ehrlichia spp.	Anaplasma spp.	Babesia gibsoni
				
Origin	Number of tests	% positive	% positive	% positive	% positive
Beijing	30	0	0	0	0
Changsha	5	0	40%	0	16.67%
Chengdu	35	0	0	0	5.56%
Chongqing	12	0	0	0	0
Fuzhou	40	0	2.50%	10%	10%
Guangzhou	48	0	2.08%	2.08%	3.64%
Hangzhou	35	2.86%	2.86%	5.71%	2.86%
Hefei	8	0	0	0	0
Jinan	10	0	0	0	10%
Nanning	14	0	7.14%	7.14%	0
Ningbo	24	0	0	0	0
Qingdao	12	0	0	0	0
Shanghai	49	2.04%	2.04%	6.12%	1.75%
Shenzhen	37	0	0	0	6.98%
Shijiazhuang	9	0	0	0	0
Taiyuan	25	0	0	0	4%
Tianjin	37	0	0	0	0
Xiamen	35	0	0	8.57%	2.86%
Xi'an	33	0	0	0	8.33%
Zhengzhou	28	0	0	0	6.67%
Total	526	0.38%	1.33%	2.66%	3.91%

*Borrelia* infection was only found in 2 out of the 20 city locations. *Ehrlichia* and *Anaplasma* infections were both found in 6 cities, while *B. gibsoni* infection was found in 12 out of 20 cities. The cities where tick-borne diseases were most frequently detected (seropositivity detected for more than two pathogens) were all located in southern cities of China including Hangzhou, Fuzhou, Guangzhou, Ximen, Shanghai, Nanning and Changsha. With the exception of Ningbo, in the 6 cities located in northern China (i.e., north of the Yangzi River), no tick-borne infections were detected.

### Identification of tick species

As presented in Table 3, a total of 1550 ticks were collected from dogs during this investigation. Except for Hefei and Chengdu, where no ticks were collected, 1 to 278 ticks were collected from the remaining 18 cities. The ticks collected were of the three development stages i.e., larval, nymphal and adult ticks, where adults, nymphs and larvae counted for 65%, 24.5% and 10.5%, respectively. All stages were identified. The species identified were *Rhipicephalus haemaphysaloides* (12.5%), *Haemaphysalis longicornis* (18.4%), and *Rhipicephalus sanguineus* (68.2%).

**Table 3:** Identification of tick samples collected from dogs.

Origin	Number of ticks	Developmental stage			Identification of species			
			
		Larva	Nymph	Adult	*Rhipicephalus haemaphysaloides*	*Rhipicephalus sanguineus*	*Haemaphysalis longicornis*	Unable to identify due to damage
Beijing	56	24	25	7		27	24	5
Changsha	71			71		71		
Chengdu	0							
Chongqing	20	20					20	
Fuzhou	133		32	101		133		
Guangzhou	278	6	10	262	195	83		
Hangzhou	249		215	34		249		
Hefei	0							
Jinan	17	9	7	1			17	
Nanning	72	4	3	65		72		
Ningbo	36		4	32		30	6	
Qingdao	14		12	2		14		
Shanghai	13		3	10		6	7	
Shenzhen	235	5	11	219		231		4
Shijiazhuang	30	14	10	6		1	29	
Taiyuan	1		1			1		
Tianjin	22		9	13		12	9	1
Xiamen	123		4	119		123		
Xi'an	29	3	25	1		5	23	1
Zhengzhou	151	78	8	65			151	
Total	1550	163	379	1008	195	1058	286	11

### Detection of pathogens carried by ticks

PCR tests were performed for 5 pathogens in 617 ticks, and sequencing was conducted to determine the pathogen species. The results are shown in Table 4. The most commonly identified infection was *Ehrlichia canis* (10.21%), followed by *Anaplasma platys* (3.4%), *B. gibsoni* (2.27%), and *Borrelia burgdorferi* (0.32%).

**Table 4:** Pathogen detection in different ticks collected from different locations.

Pathogen	Tick species (No. positive/No. samples)	Positivity	Location of positive samples (No. positive)
*Babesia canis*	*R. sanguineus (0/453)* *H. longicornis(0/91)* *R. haemaphysaloides (0/73)*	0 0 0	
*Babesia gibsoni*	*R. sanguineus (8/453)* *H. longicornis(5/91)* *R. haemaphysaloides (1/73)*	1.77% 5.49%^a^ 1.37%	Fuzhou (1), Guangzhou (1), Xiamen (1), Beijing (4), Taiyuan (1) Beijing (2), Xi'an (3) Guangzhou (1)
*Ehrlichia canis*	*R. sanguineus (50/453)* *H. longicornis(3/91)* *R. haemaphysaloides (10/73)*	11.03% 3.29%^a^ 13.69%	Hangzhou (9), Fuzhou (6), Guangzhou (9), Shenzhen (20), Nanning (3), Qingdao (1), Ningbo (1), Changsha (1) Shijiazhuang (3) Guangzhou (10)
*A.* *Anaplasma platys*	*R. sanguineus (12/453)* *H. longicornis(7/91)* *R. haemaphysaloides (2/73)*	2.65% 7.69%^a^ 2.74%	Hangzhou (3), Guangzhou (3), Shenzhen (2), Nanning (3), Qingdao (1) Zhenzhou (7) Guangzhou (2)
*Borrelia burgdorferi*	*R. sanguineus (2/453)* *H. longicornis(0/91)* *R. haemaphysaloides (0/73)*	4.4% 0 0	Hangzhou (2)

a Statistically significant (*p* value < 0.05).

The pathogens detected in different ticks are shown in Table 4: *B. gibsoni* and *A. platys* were mostly found in the tick *H. longicornis,* but *E. canis* was predominantly found in *R. haemaphysaloides* and *R. sanguineus. B. burgdorferi* was only found in the tick *R. sanguineus.* The statistical analysis indicated that *B. gibsoni,* *A. platys, E. canis,* and *B. burgdorferi* infections in the tick *H. longicornis* were significantly different from those in the ticks *R. haemaphysaloides* and *R. sanguineus*.

### Co-infection of pathogens in ticks

Ticks co-infected with different pathogens are shown in Table 5. One *R. sanguineus* tick was found to be co-infected with three pathogens *(E. canis, A. platys,* and *B. burgdorferi).* Frequent co-infections with *E. canis* and *A. platys* were observed in *R. haemaphysaloides* and *R. sanguineus.* No co-infections were observed in the tick *H. longicornis*.

**Table 5:** Co-infection with pathogens in ticks in this study.

Tick species	No. (%) of ticks infected with			
	
	Two pathogens		Three pathogens	
		
	Bg + Ec	Bg + Ap	Ec + Ap	Ec + Ap + Bb
*Rhipicephalus sanguineus* (n = 453)	1 (0.22%)	1 (0.22%)	5 (1.10%)	1 (0.22%)
*Haemaphysalis longicornis*				
(n = 91)	0	0	0	0
*Rhipicephalus haemaphysaloides* (n = 73)	1 (1.37%)	0	1 (1.37%)	0
Total (n = 617)	2 (0.32%)	1 (0.16%)	6 (0.97%)	1 (0.16%)

Bg*: B. gibsoni*; Ec: *E. canis*; Ap: *A. platys*; Bb: *B. burgdorferi.*

## Discussion

Ticks and tick-borne diseases in owned pet dogs from 20 large Chinese cities were investigated. A large number of samples from various locations were collected. This is the first large-scale investigation of ticks and tick-borne pathogens in pet dogs and it revealed a wide distribution of ticks and pathogens, and thus the risk of vector-borne disease. Tick-borne diseases were mainly identified in southern China, which confirms that the distribution of tick-borne diseases is geographical in nature.

*B. burgdorferi* is the agent of Lyme disease, which occurs globally, and can infect a wide-range of animals including rodents, ruminants, carnivores, and birds, as well as humans. Among samples from 526 pet dogs, 0.38% were serologically positive for *Borrelia* infection, which correlates with investigations performed in dogs in individual reports in other countries [15,16].Considering the vector's geographical distribution and abundance, it is easy to understand why the rate of positive samples reported here was significantly lower than the 4.5–11% and 1.4–11.6% infection rates reported in dogs in the UK and USA, respectively [17,18]. Lyme disease was first reported in China in 1985 with a seropositivity rate of 1.06∼12.8% in the 30 000 people randomly sampled [19]. In contrast, *Borrelia* infections in dogs appear to be less common than in humans, with only a single positive sample found in 300 serological samples from Beijing [10]. To the best of the authors' knowledge, no other reports utilizing serological or molecular methods present data on *Borrelia* infections in dogs in China. Our data indicate that the infection rate with *Borrelia* in pet dogs in south-eastern China is low.

The two ticks collected from pet dogs that were PCR-positive for *Borrelia* were identified as *R. sanguineus* and were both from Hangzhou. It is commonly considered that only *Ixodes* is a vector for *Borrelia*, but no *Ixodes* spp. were collected during this survey. It had been reported that *H. longicornis* and *R*. *haemaphysaloides* ticks could carry *Borrelia* in China [20], but no reports are available for *R. sanguineus* acting as a carrier. The possibility exists that *R. sanguineus* may have ingested *Borrelia* from infected dogs, but this does not necessarily qualify the tick as a vector. Only two dogs were found serologically positive for *Borrelia* infection; they were located in the Hangzhou and Shanghai areas which are approximately 180 kilometres apart and thus in relative geographic proximity to each other. This finding warrants further study on the prevalence of *Borrelia* and its tick-borne vector(s).

Ehrlichiosis and anaplasmosis are emerging tick-borne diseases in both humans and animals. *E. canis* and *A. platys* are the two best known pathogens that cause canine ehrlichiosis and anaplasmosis. Both agents have a worldwide distribution and were thought to be transmitted by *R. sanguineus* [21]. In this survey, serological tests from 526 pet dog samples demonstrated a rate of 1.33% for *E. canis* infection and 2.66% for *Anaplasma* spp. infection. Preliminary studies indicate that *A. phagocytophilum* antigens in SNAP^®^ 4Dx^®^ cross-react with samples from *A. platys*-infected dogs (SNAP^®^ 4Dx^®^ kit insert 06-28502-08 IDEXX Laboratories 2017). Similar serological evaluation demonstrated a high infection rate for *E. canis* infection and for *Anaplasma* spp. infection in dogs in other countries [22,16]. The overall annual incidence of canine ehrlichiosis was estimated to be 2.1 cases per thousand dogs in France [23]. In the United States, canine ehrlichiosis is a sporadic disease [24]. A high prevalence (36%) of active infection was recently detected in dogs infested by *R. sanguineus* in north-eastern Arizona [25]. In China, serological and PCR-based study results for *Ehrlichia* and *Anaplasma* infection have been reported concerning ticks, animals and humans [26,27,28,29]. This study reports the first detection of *H. longicornis* and *R*. *haemaphysaloides* as vectors of *E. canis* and *A. platys.* The three commonly identified tick species (*R. sanguineus, H. longicornis* and *R*. *haemaphysaloides*) demonstrated a high infection rate for both *E. canis*, and *A. platys*. Based on the number of dogs sampled and their distribution, we cannot define the results as prevalences but observed infection rates. Nevertheless, the infection rates identified in this study were closely related to the serological prevalence observed in dogs in other published studies mentioned above. Particular attention should be paid to their presence due to their zoonotic potential [2,30].

*B. gibsoni* is a virulent protozoan parasite of dogs and is one of the most important tick-borne diseases of domestic dogs. In this study, the ELISA test demonstrated an infection rate for *B. gibsoni* of 3.91% in pet dogs, which is similar to the seroprevalence reported in pet dogs in East China of 3.47% [7]. *B. gibsoni* is transmitted by ticks including *H. longicornis* [31] and *R. sanguineus* [32]. This survey also showed that *B. gibsoni* could be detected in *R.* *haemaphysaloides* ticks in China. Although the tick *R. haemaphysaloides* was found to carry *B. gibsoni* in this study, further studies will be necessary to clarify its actual potential as a vector of the pathogen.

The results of identification of tick species are consistent with previous studies indicating that *R. sanguineus*, *H. longicornis* and *R*.* haemaphysaloides* are the predominant species infesting pet dogs in China [7]. Here, larval, nymphal and adult stages were identified on pet dogs. In this study, *B. canis* was not detected in ticks. Ticks co-infected with multiple pathogens were found in this survey, which increases the risk of co-infections in both dogs and humans. Co-infections might result in more complex clinical manifestations and could complicate the possible diagnosis of the infecting pathogen. As yet, there are no reports of co-infections with tick-borne pathogens in humans in China; however, concerns have been raised because the pathogens might share common tick vectors and reservoir hosts, which means transmission of co-infections to humans may indeed be possible.

Owing to sampling limitations, this report provides only estimates of infection rates of important tick-borne diseases in dogs. However, the information revealed in this study confirms the correlation between ticks and the canine tick-borne diseases. Given the threat posed by ticks to dogs and the zoonotic implications of tick infestations in dogs, the critical need for effective treatment and routine prevention of tick infestations in dogs is emphasized by the findings of this study.

## Conflict of interest

The work reported herein was partially funded by Merial. Several authors were employees or contractors of Merial.
